# Serum vascular endothelial growth factor-C levels: A possible diagnostic marker for lymph node metastasis in patients with primary non-small cell lung cancer

**DOI:** 10.3892/ol.2013.1373

**Published:** 2013-06-04

**Authors:** YAKUN ZHANG, XUE MENG, HONGSHENG ZENG, YAN GUAN, QIONG ZHANG, SHEN GUO, XIUJIU LIU, QISEN GUO

**Affiliations:** 1 Departments of Medical Oncology, Jinan, Shandong 250117, P.R. China; 2 Radiation Oncology, Shandong Cancer Hospital, Shandong Academy of Medical Sciences, Jinan, Shandong 250117, P.R. China

**Keywords:** vascular endothelial growth factor-C, lymph node metastasis, enzyme-linked immunosorbent assay, non-small cell lung cancer, polymerase chain reaction, diagnosis

## Abstract

Accurate tumor staging is essential for selecting the appropriate treatment strategy for lung cancer. Computed tomography (CT), or positron emission tomography (PET), is the most commonly used non-invasive staging method of lymph node (LN) metastases (LNM), but this method remains unsatisfactory. The present study measured vascular endothelial growth factor (VEGF)-C levels in serum, tumor tissue and LNs to determine the correlation between serum VEGF-C and LNM, and also assessed the usefulness of serum VEGF-C as an additional diagnostic marker for identifying LNM. A total of 66 patients with non-small cell lung carcinoma (NSCLC) or benign tumors of the lung were included in this study, and circulating VEGF-C levels were assessed with enzyme-linked immunosorbent assays. RNA fractions extracted from the tumor tissues and LNs were subjected to quantitative polymerase chain reaction (qPCR) to assess the mRNA levels of VEGF-C. The VEGF-C levels in serum, tumor tissue and LNM were significantly higher compared with the control group (P<0.05). The VEGF-C levels of patients with LNM were significantly higher compared with those without LNM (P<0.05). The VEGF-C levels in the serum, tumor tissue and LNM were significantly correlated (P<0.05). With regard to the diagnosis of LNM using VEGF-C levels, the serum levels of VEGF-C reached a sensitivity of 65.0% and a specificity of 72.2% when a cutoff value of 655.65 pg/ml was applied. Serum VEGF-C levels may provide additional information for distinguishing between the absence and presence of LNM in patients with lung carcinoma. The evaluation of serum VEGF-C is complementary to accurate LN staging in NSCLC.

## Introduction

The accurate staging of lymph node (LN) metastases (LNM) is critical for determining the optimal treatment strategy for patients with non-small cell lung cancer (NSCLC). Computed tomography (CT), or positron emission tomography (PET), is the most commonly used non-invasive staging method of LNM. The CT imaging criteria for tumor involvement rely on the size and shape of the LNs. However, even when this size is <1 cm, the rate of LNM is 10% (1). Certain studies have demonstrated that vascular endothelial growth factor (VEGF)-C is a major factor associated with the growth of lymphatic endothelial cells (2–3). It has also been observed that the expression of VEGF-C in tumor tissue is significantly associated with LNM, lymphatic vessel invasion and, furthermore, nodal microdissemination (4–5). However, compared with examining surgically obtained tissue specimens, serum assays may be performed easily and frequently due to their minimal invasiveness. In the present study, the correlation between circulating VEGF-C levels and pathologically proven LNM was analyzed and an evaluation was made as to whether circulating VEGF-C was able to provide additional information for discriminating between the absence and presence of LNM in patients with lung cancer.

## Materials and methods

### Patients

Between January 2007 and October 2009, 66 patients underwent surgery for primary tumors of the lung at Shandong Cancer Hospital (Shandong Academy of Medical Sciences, Jinan, Shandong, China). Peripheral venous blood samples were obtained prior to surgery from 56 patients with primary NSCLC and 10 patients with benign tumors of the lung. All patients underwent diagnostic procedures prior to surgery using brain and body CT scans and bone scintiscans. The present study was conducted according to the institutional and ethics rules concerning research on tissue specimens and the study was approved by the ethics committee of Shandong Cancer Hospital, Shandong, China. Written informed consent was obtained from all patients. A total of 56 patients with NSCLC received curative surgery with routine systematic nodal dissection of the hilar and mediastinal LNs. The pathological stage was classified as stage I in 16 patients, stage II in 17 patients and stage III in 23 patients. The histopathological types included 26 adenocarcinomas, 24 squamous cell carcinomas and 6 adenosquamous cell and large cell carcinomas. The characteristics of the 56 patients are shown in Table I. No patients received blood transfusions, radiotherapy or chemotherapy prior to the study.

### Measurement of VEGF-C levels in blood samples

Blood samples were drawn pre-operatively by venous puncture and divided into plain tubes without anticoagulant for the serum. Within 1 h of collection, the blood samples were centrifuged at 778 × g for 10 min within 1 h of collection and the aliquots were frozen at −80°C for later analysis. The VEGF-C kit was provided by Adlitteram Diagnostic Laboratories (San Diego, CA, USA). VEGF was assayed using commercially available sandwich enzyme-linked immunosorbent assay kits (Adlitteram Diagnostic Laboratories) according to the manufacturer’s instructions. The sensitivity limit of the VEGF-C assays was 0.1 ng/ml. The coefficient of variation was <5.0%.

### Measurement of VEGF-C levels in tumor and LN samples

Fresh tissues were snap frozen in liquid nitrogen and stored at −80°C until use. During the analyses, extracts were made from the tissue and LN samples and the mRNA levels of the extracts were analyzed. The extraction method involved the following: Total RNA was extracted using the TRIzol method (Invitrogen, Carlsbad, CA, USA) according to the manufacturer’s instructions. The purity and concentration of the total RNA was then determined with spectrophotometry at 260 and 280 nm. Total RNA was reverse transcribed to cDNA using a First-Strand cDNA Synthesis kit (Takara, Otsu, Japan). To confirm RNA quality, the RT products were checked with PCR using a pair of primers specific for GAPDH. No significant degradation was observed in any of the RNA samples.

The quantitative polymerase chain reaction (qPCR) was performed with a QuantiTect SYBR Green PCR kit (Takara). A cDNA pool serially diluted from 1:10 to 1:1,000 was used to generate standard curves. The data were normalized to the housekeeping GAPDH gene. The protocol of the PCR was as follows: Incubation at 95°C for 10 min followed by 40 cycles, which included preliminary denaturing at 95°C for 10 sec, annealing at 55°C for 10 sec and extension at 72°C for 15 sec. All reactions were performed in triplicate. The PCR was evaluated by melting curve analysis and the calculations for determining the relative level of gene expression were performed using the cycle threshold (Ct) method. The mean Ct values from the triplicate measurements were used to calculate the relative expression of the target genes with normalization to GAPDH (used as an internal control) via the 2^−ΔΔCt^ method. Primers were synthesized by Shanghai Sangon Biological Engineering Technology & Services Co., Ltd. (Shanghai, China) as follows: VEGF-C forward, 5′-TCAAGGACAGAA GAGACTATAAAATTTGC-3′ and reverse, 5′-ACTCCAAAC TCCTTCCCCACAT-3′; GAPDH forward, 5′-CAACAGCCT CAAGATCATCAGC-3′ and reverse, 5′-TTCTAGACGGCA GGTCAGGTC-3′.

### Statistical analysis

Statistical analysis was performed with the SPSS statistical software package, version 10.0 (SPSS Inc., Chicago, IL, USA). Differences in distribution were determined using t-tests. The associations between the levels of VEGF-C and the clinicopathological features were evaluated using the Spearman’s rank correlation coefficient of Fisher’s exact probability test. P<0.05 was considered to indicate a statistically significant difference.

## Results

The median serum VEGF-C concentration (25–75th quartile) was 655.7±103.6 pg/ml (612.7–762.4 pg/ml) in patients with lung carcinoma and 577.5±44.2 pg/ml (547.5–586.8 pg/ml) in patients with benign tumors. These concentrations were significantly different (P=0.012). The correlation between the clinicopathological findings and the expression of VEGF-C at the serum, tumor tissue and LN levels are shown in Table I. Of the 56 patients with NSCLC, 38 patients had metastatic LNs while 18 had no LNM. Patients with LNM exhibited higher serum VEGF-C levels compared with those without (697.7±96.9 pg/ml vs. 532.5±95.9 pg/ml, respectively; P=0.026). Similarly, significant differences were detected between the different clinical stages (stage I, 623.2±109.6 pg/ml vs. stage II, 632.1±126.5 pg/ml vs. stage III, 712.2±107.4 pg/ml, respectively; P=0.017). Serum VEGF-C concentration exhibited a trend of gradually increasing with histological grade, but a statistically significant difference was not detected (P=0.512). There were no associations between the expression of VEGF-C in serum and the patient age, gender, histology, histological grade or tumor size (P>0.05).

In addition, the median mRNA level of VEGF-C in the cytoplasm of tumor cells was 59.6±12.5 in patients with lung carcinoma and 42.8±8.5 in patients with benign tumors. These concentrations were statistically significantly different (P=0.001). Among the 56 patients with NSCLC, high VEGF-C mRNA levels in the lung tumor tissue cells were associated with a significantly higher incidence of LNM. The median mRNA levels of VEGF-C in primary tumor tissues with and without LNM were 62.3±15.3 and 48.2±12.6, respectively (P=0.001). Similarly, the median VEGF-C mRNA level in LNM was 61.1±14.2, while it was 49.5±12.1 in patients without LNM (P=0.004). There was no association between VEGF-C mRNA levels in tumor tissue or LNs and the patient age, gender, histology, histological grade and tumor size (P>0.05).

**Figure 1.**
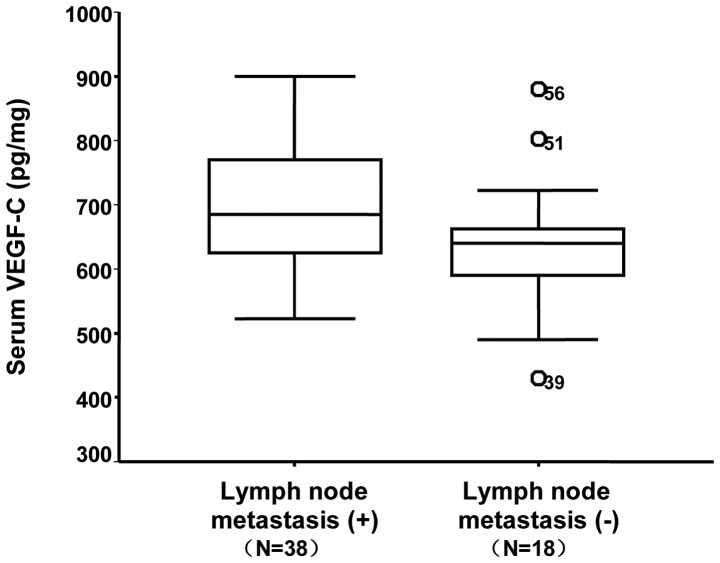
Box plot representation of serum VEGF-C levels in patients with lung cancer, with regard to lymph node metastasis (LNM). Patients with LNM revealed higher serum VEGF-C concentrations than those without (P=0.026). VEGF-C, vascular endothelial growth factor-C.

**Figure 2.**
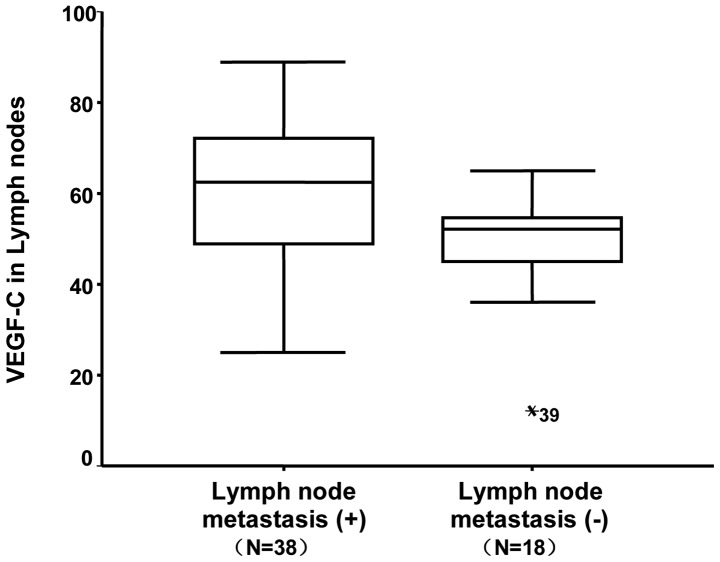
Box plot representation of VEGF-C levels in lymph nodes (LNs) in patients with lung cancer, with regard to lymph node metastasis (LNM). Patients with LNM revealed higher VEGF-C concentrations than those without (P=0.004). VEGF-C, vascular endothelial growth factor-C.

**Figure 3.**
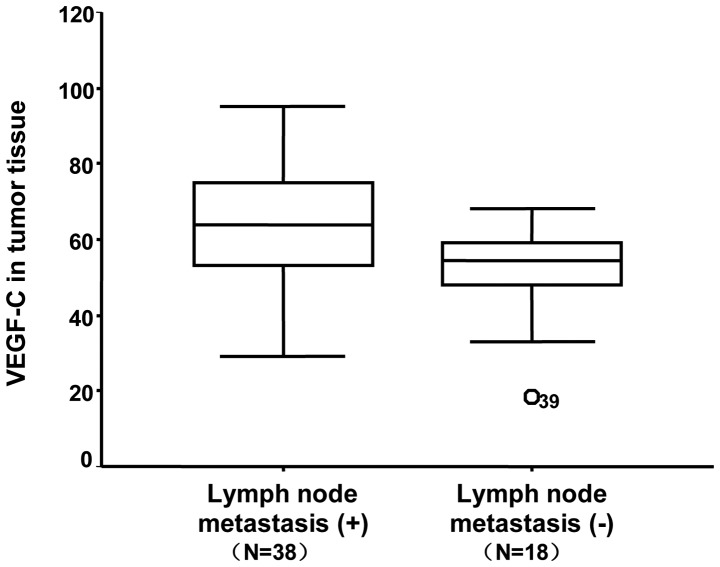
Box plot representation of VEGF-C levels in tumor tissue in patients with lung cancer, with regard to lymph node metastasis (LNM). Patients with LNM revealed higher VEGF-C concentrations than those without (P=0.001). VEGF-C, vascular endothelial growth factor-C.

By linear correlation analysis, there was a positive correlation between the VEGF-C level of peripheral blood and that of tumor tissue with NSCLC (r=0.629, P<0.001). An association was observed between the VEGF-C levels in the LNs and peripheral blood of patients with NSCLC (r=0.755, P<0.001), as well as between the levels in the LNs and tumor tissue, which were positively correlated (r=0.838, P<0.001). All cases were classified into three groups according to the VEGF-C levels in the serum, LNs and tumor tissue, and the median VEGF-C concentrations in the LNM group were 697.7±96.9, 61.1±14.2 and 62.3±15.3 pg/ml in the serum (Fig. 1), LNs (Fig. 2) and tumor tissue (Fig. 3), respectively. With regard to the diagnosis of LNM using VEGF-C levels, the VEGF-C serum levels reached a sensitivity of 65.0% and a specificity of 72.2% when a cutoff value of 655.65 pg/ml was applied.

## Discussion

The present study used VEGF-C levels in serum, tumor tissue and LNs to determine the correlation between circulating VEGF-C levels and LNM. In the present study, a clear and significant correlation was observed between VEGF-C levels and LNM. These results were in keeping with those of Masaya *et al* (6). Moreover, NSCLC patients with LNM had higher VEGF-C levels in the cytoplasm of tumor cells compared with those without metastasis. The VEGF-C concentrations in LNM were significantly higher compared with those without LNM. There were positive correlations between the VEGF-C levels of peripheral blood and tumor tissues and LNM from the NSCLC samples. Notably, the present study revealed that although significant differences were detected between the different clinical stages, the serum VEGF-C levels were not significantly associated with tumor size.

Accurate tumor staging is essential for selecting the appropriate treatment strategy for patients with cancer in general, but in particular for patients with lung carcinoma. The involvement of the mediastinal LNs is a significant prognostic factor in patients with potentially resectable NSCLC. Surgical techniques, such as mediastinoscopy or endobronchial ultrasound guided transbronchial needle aspiration (EBUS), are widely regarded as the most useful methods for mediastinal staging (7). Non-invasive imaging studies, such as CT and magnetic resonance imaging (MRI) scans, are less reliable since the imaging criteria for tumor involvement are morphological, relying on the size and shape of the LNs. With regard to identifying the presence or absence of LNM, the accuracy of CT has been reported to be between 51.4 and 83.0% (8,9). Several studies have described ^18^F-fluorodeoxyglucose (FDG)-PET as being advantageous for diagnosing the LN staging of NSCLC. The sensitivity and specificity of PET have been reported to be 67–89% and 82–99%, respectively (10–12). A non-invasive, accurate and easily performed technique for LN staging is urgently required as surgical techniques are invasive and PET is performed only at a limited number of facilities. The evaluation of serum VEGF-C concentrations would be useful for hospitals where there is no access to PET scanning; furthermore, it is a non-invasive and inexpensive examination technique. In the present study, with regard to the diagnosis of LNM using VEGF-C levels in serum, a cutoff value of 655.65 pg/ml was applied

The predictive value of CT in diagnosing LNM is affected by biases due to LN size and shape. Using a combination of VEGF-C assays and CT or PET may result in suitable positive and negative predictive values for this diagnosis. Serum VEGF-C levels may be used as an excellent complementary approach for obtaining a high sensitivity and specificity. This is likely to contribute to the selection of patients and avoid unnecessary surgery. It should be noted that a combined diagnosis by serum VEGF-C levels and CT or PET is relatively accurate, although there are false-positive as well as false-negative cases. It may be dangerous to start induction chemotherapy relying solely on VEGF-C levels. Invasive staging such as mediastinoscopy or EBUS cannot be omitted, but we propose that the presented combined diagnosis provides useful information for selecting patients who require or do not require mediastinoscopy. In the future, in order to examine the diagnostic value of a combined assay using other markers, a more detailed study is required.

In conclusion, the present study demonstrated that serum VEGF-C levels provide additional and useful information for discriminating between the absence and presence of LNM in patients with lung carcinoma. These findings suggest that VEGF-C may be an ideal target for diagnosis or therapy to improve the prognosis of patients with this deadly disease. The pre-operative evaluation of serum VEGF-C concentrations in patients with primary NSCLC is non-invasive, easily performed and inexpensive. Making a combined diagnosis with serum VEGF-C evaluation is a more reliable marker. Further investigation is necessary to determine and understand the role of VEGF-C in patients with NSCLC. In the future, to examine the diagnostic value of serum VEGF-C levels for predicting LNM microdissemination and to compare its diagnostic utility with that of commonly used tools such as CT, MRI and FDG-PET scans, a more detailed study is required.

## Figures and Tables

**Table I.** Associations between clinicopathological findings and expression of VEGF-C in patients with primary lung carcinoma.

Characteristics	No.	Serum VEGF-C levels (pg/ml)		LN VEGF-C levels (mRNA)		Tumor VEGF-C levels (mRNA)	
		
		Concentration	P-value	Concentration	P-value	Concentration	P-value
Age (years)			0.600		0.452		0.302
<60	20	661.5±110.1		59.2±15.9		58.1±16.6	
≥60	36	653.2±99.9		56.2±14.3		52.3±17.4	
Gender			0.660		0.345		0.409
Male	46	653.2±102.8		58.2±14.6		54.8±16.2	
Female	10	676.5±111.5		53.4±13.7		50.2±11.8	
Tumor histology			0.793		0.562		0.566
Adenocarcinoma	26	679.4±124.1		46.3±11.6		47.9±12.6	
Squamous cell carcinoma	24	672.8±110.6		52.5±13.7		53.4±13.7	
Other	6	645.2±139.5		51.5±19.8		50.9±19.8	
Histological grade			0.512		0.213		0.205
Well-differentiated	11	627.2±121.0		51.1±13.8		52.1±13.8	
Moderately-differentiated	24	685.2±113.5		53.3±14.7		54.8±14.2	
Poorly-differentiated	21	719.3±111.0		54.9±13.4		56.7±13.5	
Tumor size			0.334		0.589		0.561
Diameter ≤3 cm	13	652.2±84.5		55.4±12.0		53.7±12.8	
Diameter >3 cm	43	684.1±108.5		57.9±15.2		59.8±16.5	
LNM			0.026		0.004		0.001
Positive	38	697.7±96.9		61.1±14.2		62.3±15.3	
Negative	18	532.5±95.9		49.5±12.1		48.2±12.6	
Stage			0.017		0.621		0.632
I	16	623.2±109.6		53.2±11.8		54.2±12.8	
II	17	632.1±126.5		55.7±12.8		56.8±13.8	
III	23	712.2±107.4		59.3±15.3		58.1±16.3	

Values for concentrations are presented as median ± SD. VEGF-C, vascular endothelial growth factor-C; LN, lymph node; LNM, lymph node metastases.

## References

[1] Miller DL, Rowland CM, Deschamps C (2002). Surgical treatment of non-small cell lung cancer 1 cm or less in diameter. Ann Thorac Surg.

[2] Nishida N, Yano H, Komai K (2004). Vascular endothelial growth factor C and vascular endothelial growth factor receptor 2 are related closely to the prognosis of patients with ovarian carcinoma. Cancer.

[3] Cianfarani F, Mastroeni S, Odorisio T (2012). Expression of vascular endothelial growth factor-C in primary cutaneous melanoma predicts sentinel lymph node positivity. J Cutan Pathol.

[4] Wang TB, Chen ZG, Wei XQ (2011). Serum vascular endothelial growth factor-C and lymphoangiogenesis are associated with the lymph node metastasis and prognosis of patients with colorectal cancer. ANZ J Surg.

[5] Acs G, Paragh G, Rakosy Z (2012). The extent of retraction clefts correlates with lymphatic vessel density and VEGF-C expression and predicts nodal metastasis and poor prognosis in early-stage breast carcinoma. Mod Pathol.

[6] Tamura M, Ohta Y (2003). Serum vascular endothelial growth factor-C level in patients with primary nonsmall cell lung carcinoma: a possible diagnostic tool for lymph node metastasis. Cancer.

[7] McNeil TM, Chamberlain JM (1966). Diagnostic anterior mediastinotomy. Ann Thorac Surg.

[8] Kitajima K, Yamasaki E, Kaji Y (2012). Comparison of DWI and PET/CT in evaluation of lymph node metastasis in uterine cancer. World J Radiol.

[9] Zhang L, Xi M, Deng XW (2012). Four-dimensional CT-based evaluation of volumetric modulated arc therapy for abdominal lymph node metastasis from hepatocellular carcinoma. J Radiat Res.

[10] Takenaka T, Yano T, Morodomi Y (2012). Prediction of true-negative lymph node metastasis in clinical IA non-small cell lung cancer by measuring standardized uptake values on positron emission tomography. Surg Today.

[11] Geraldson CT, Stephenson JE, Lagrew JP (2012). Use of positron emission tomography in initial staging of nonsmall cell lung carcinoma: a regional teaching hospital experience. Am Surg.

[12] Kernstine KH, Mclaughlin KA, Menda Y (2002). Can FDG-PET reduce the need for mediastinoscopy in potentially resectable nonsmall cell lung cancer?. Ann Thorac Surg.

